# Real-Time Prediction of Joint Forces by Motion Capture and Machine Learning

**DOI:** 10.3390/s20236933

**Published:** 2020-12-04

**Authors:** Georgios Giarmatzis, Evangelia I. Zacharaki, Konstantinos Moustakas

**Affiliations:** VVR Group, Department of Electrical and Computer Engineering, University of Patras, 26504 Patras, Greece; ezachar@upatras.gr (E.I.Z.); moustakas@upatras.gr (K.M.)

**Keywords:** contact force prediction, musculoskeletal modeling, support vector regression, artificial neural networks, gait analysis

## Abstract

Conventional biomechanical modelling approaches involve the solution of large systems of equations that encode the complex mathematical representation of human motion and skeletal structure. To improve stability and computational speed, being a common bottleneck in current approaches, we apply machine learning to train surrogate models and to predict in near real-time, previously calculated medial and lateral knee contact forces (KCFs) of 54 young and elderly participants during treadmill walking in a speed range of 3 to 7 km/h. Predictions are obtained by fusing optical motion capture and musculoskeletal modeling-derived kinematic and force variables, into regression models using artificial neural networks (ANNs) and support vector regression (SVR). Training schemes included either data from all subjects (*LeaveTrialsOut*) or only from a portion of them (*LeaveSubjectsOut*), in combination with inclusion of ground reaction forces (GRFs) in the dataset or not. Results identify ANNs as the best-performing predictor of KCFs, both in terms of Pearson *R* (0.89–0.98 for *LeaveTrialsOut* and 0.45–0.85 for *LeaveSubjectsOut*) and percentage normalized root mean square error (0.67–2.35 for *LeaveTrialsOut* and 1.6–5.39 for *LeaveSubjectsOut*). When GRFs were omitted from the dataset, no substantial decrease in prediction power of both models was observed. Our findings showcase the strength of ANNs to predict simultaneously multi-component KCF during walking at different speeds—even in the absence of GRFs—particularly applicable in real-time applications that make use of knee loading conditions to guide and treat patients.

## 1. Introduction

Musculoskeletal diseases, along with natural age-related sensorimotor decline, affect the lower limbs’ soft tissue homeostasis and/or skeletal integrity, resulting in pain [1], muscle loss [2] and functional decline [3,4] along with elevated fall [5,6] and fracture risk [7]. Even though knowledge discovery has been immense during recent decades, the scientific community has been unsuccessful in treating musculoskeletal diseases, and current prevalence, incidence, and socioeconomic burden still impose a significant threat to healthcare systems [8,9]. Measures to counter their adverse effects include pharmacological and rehabilitation interventions, mainly utilized when health status has already diminished, and the patient seeks medical help. On the other hand, exercise has proven a lifetime successful surrogate strategy for prevention and treatment of numerous pathologies [10], since it can trigger an anabolic response to muscle and bone matrix and improve neuromotor function, followed by improvement in key cardiovascular indicators [11] and overall quality of life [12]. Research interest has focused on characterizing the mechanical loading exerted during exercise in important skeletal sites, in order to elucidate the interplay between external forces and biological response of the human skeleton [13,14,15].

Forces acting on joints and resulting bone deformation (strain) can lead to degeneration of impact-absorbing cartilage [16] or regulate fracture-protective bone mass and shape [15]; hence, their exploration constitutes an urgent matter for prevention of major diseases, such as osteoarthritis (OA) and osteoporosis. Important descriptors of joint loading are joint contact forces, which are highly affected by individual motion strategy [17,18,19,20], aberrant bone geometry [21,22], and are also related to cartilage stress distribution [23,24,25] and joint osteoarthritis initiation and progression [26,27]. As a result, the joint contact forces have been extensively used to assess joint malfunction [28], disease-driven gait alterations [29] and joint replacement design [30,31]. Medial knee force calculation has particularly attracted research efforts due to the high prevalence of medial knee osteoarthritis compared to the lateral compartment [32], thus medial knee contact force (KCF) reduction has been the focus of numerous gait modification regimens [33,34]. However, knowledge of inter-compartmental knee loading patterns and force direction during locomotion will uplift the design of knee rehabilitation programs for lateral osteoarthritis patients as well, and help towards the investigation of the mechanical factors of knee prostheses’ loosening phenomenon [35]. 

The lack of actual forces/strains acting on bone, caused due to the invasiveness of available acquisition techniques [36,37], has led to their estimation via modeling algorithms of muscle function and bone mechanical response. Musculoskeletal models provide validated, clinically relevant, subject-specific information, although their demand for high expertise, comprehensive collection of input variables and excessive computational cost has inhibited their widespread use in the clinical world. Moreover, standard modeling methods can be rather time-consuming—ranging from several minutes to hours—thus being unsuitable for real-time applications. A representative example involves the assessment and guidance of knee-osteoarthritic patients in self-managing their condition through medial knee offloading while performing daily activities [33,34]. Consequently, new approaches must emerge to facilitate prevention and treatment of major musculoskeletal deficits, integrating sensor-based biomedical technologies coupled with computational models of muscle and bone.

As an alternative to biophysics-driven modeling approaches, data-driven techniques are rapidly evolving to meet healthcare needs. The advancement of sensing devices that allow one to monitor health-related parameters [38], as well as the increase in computation capacity permitting real-time simulation and modelling of human body functions [39], has opened numerous opportunities for machine learning (ML) in healthcare. ML methods’ strength lies on their ability to leverage non-linear relationships between a set of inputs and outputs and build a model to “learn” the interaction between them through a rigorous training phase on—usually large—acquired datasets. Once the formulation and parameters of the model have been selected, it can offer predictions very fast, highlighting ML’s usability as tool to predict key biomechanical variables. ML-based models can easily be embedded in digital tools to become suitable for real-time applications.

Artificial neural networks are commonly used to predict lower limb joint angles and moments [40,41,42,43,44], ground reaction forces [45], joint forces or impulses [46,47,48,49] and contact pressures [50,51,52]. On the other hand, support vector machines have also demonstrated promising prediction performance in various regression biomechanical problems, such as electromyography (EMG)-based prediction of lumbosacral joint loads [53] and optical marker-based prediction of lower limb joint angles and moments [54,55], standing out for their substantial generalization ability to unseen datasets [56]. For an analytic review on ML applications in human movement biomechanics, including also unsupervised learning techniques, we refer to the survey of Halilaj et al. [57]. Since a trained ML model is as good as its training dataset, and most studies have used a very limited dataset of patient population or limited range of walking speeds [47,48], generalization of their predictions may be restricted. In most cases, input datasets were related to the motion capture (mocap) system used for motion analysis, for example, the original trajectories from markers or inertial measurement unit (IMU) signal were primarily included, thus limiting the usage of the trained models beyond the specific marker set protocol or IMU placement and signal processing. Finally, the output variables were usually restricted to single medial KCF [47,48], and no multi output regression techniques were utilized to promote understanding of overall, multi-component knee joint loading. 

Thus, the aim of this paper is to establish an ML-empowered, mocap-agnostic framework to predict all components of medial and lateral KCFs during different gait speeds, based on data fusion of calculated variables through motion analysis and musculoskeletal modelling. The predictive capability of two common regression ML methods—artificial neural network (ANN) and support vector regression (SVR)—will be researched to elucidate their applicability on prediction of important biomechanical parameters in real time. 

## 2. Experimental Procedure and Biomechanical Simulations

### 2.1. Participants

Fifty-four (54) healthy, university students and community-dwelling citizens of two distinct age groups—young (n = 40) and elderly (n = 14), (see Table 1)—provided informed consent to participate in the study, approved by the local Ethics Committee. None of them had prior lower-limb defect that could affect their performance. 

### 2.2. Experimental Protocol

**Figure 1:**
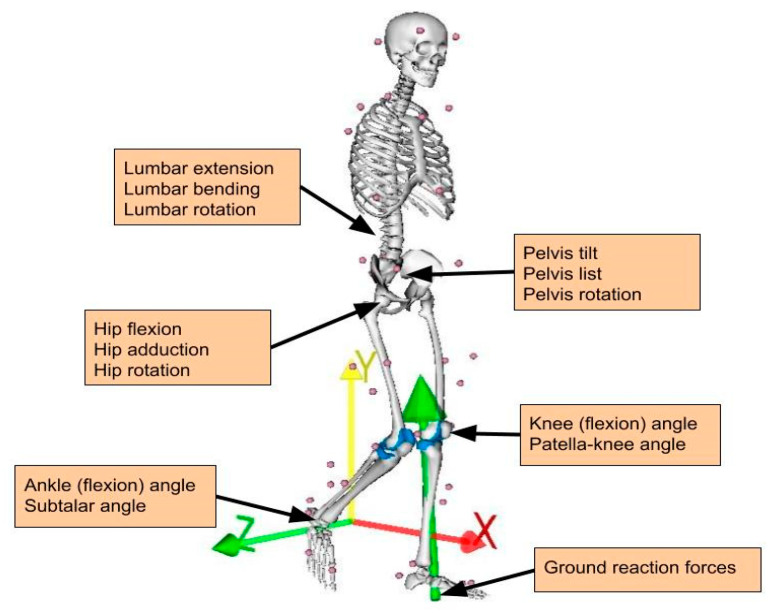
Marker set used for motion capture and joint angle/external force definition.

All participants walked on a motor driven treadmill (Forcelink, Culemborg, The Netherlands) at a speed increasing from 3 to 7 km/h, with an increment of 1 km/h. A harness attached to the ceiling was utilized to ensure participants’ safety. Subjects were advised to walk casually and choose their last walking speed on their own. They stopped at a different maximum walking speed; 4 subjects walked up to 4 km/h, 13 subjects walked up to 5 km/h, 31 subjects walked up to 6 km/h and only 5 walked up to 7 km/h. Twenty walked only barefoot, eleven walked with both shoes and barefoot interchangeably and twenty five walked only with shoes. Due to continuous recordings between speeds and the lack of an automated way to count steps, data were collected for 10 s per speed following an adaptation period of 10 s, resulting in multiple gait cycles per subject and speed. More time for acclimation was given to the elderly group, but not more than 1 min, to avoid fatigue. Forty-two (42) retroreflective markers were placed on anatomical landmarks (Figure 1) and their spatial trajectories were recorded using a 10-camera VICON system (10–15 MX camera system, VICON, Oxford Metrics, Oxford, UK) with sampling rate of 100 Hz. Ground reaction forces (GRFs) and moments were captured through force plates integrated in the treadmill at 1000 Hz and filtered at 6 Hz. Clusters of 3 markers were placed on the femur and tibia of each side to improve capturing of respective body segment motion. 

### 2.3. Musculoskeletal Modeling

The entire musculoskeletal procedure was implemented with the free open-source software, OpenSim (Stanford University, Stanford, CA, USA). First, a generic musculoskeletal model [58] consisting of 18 segments and 92 Hill-type musculotendon actuators was linearly scaled to each participant’s anatomy and body weight, based on recorded marker positions during a static trial while each participant was standing on a force plate in a neutral position. The included knee joint was modeled as a single degree of freedom (DOF) joint acting along the sagittal plane, specifying two distinct contact points between the femur and tibia lying on the medial and lateral compartment of a tibial plateau. Two revolute joints acting on the frontal plane were attached to each contact point, respectively, sharing the forces acting between the femur and tibia. Inter-compartmental forces were calculated via balancing the net reaction forces and frontal-plane torques around the knee joint. The distance between the contact points and femur–tibia alignment was kept at the default values (3.5 mm and 0, respectively) since relevant subject-specific information was not available (e.g., through medical imaging). The ankle and subtalar joints were modelled as revolute joints, whereas the hip was modelled as a ball and socket joint. Joint angles were calculated for every recorded motion using the inverse kinematics tool and, after low-pass filtering at 6 Hz, served as inputs—coupled with GRFs—to static optimization for muscle force estimation. The cost function was set to minimize the squared sum of all muscle activations. Reserve actuators were added at each joint of the model, to provide the additional torques if needed during static optimization, although their usage was penalized to avoid excessive activation. Finally, three medial and three lateral components of knee contact forces were calculated in the local coordinate system, using the calculated joint angles and muscle forces, along with the three components of GRFs, vertical GRF moment and two-dimensional coordinates of the center-of-pressure acting at each foot, as inputs to the Joint Reaction Analysis in OpenSim. These KCFs form the response variables that were subsequently used to train a regression model that takes as input the kinematic variables and the GRFs. Once trained, the model can be used for real-time prediction on new coming data. 

### 2.4. Data Pre-Processing

Individual gait cycle was determined as the stance phase of each leg and obtained between heel-strike and following ipsilateral toe-off. Respective joint angles, GRFs and calculated KCFs of each leg were synchronized by downsampling the GRFs to match the frequency of the other two. All forces were normalized to individual body weight to account for inter-subject variability and represent the residual force variation per weight unit. To include both right and left leg data in the dataset, left mediolateral component of GRFs and KCFs was negated to match the right forces. No negation was needed for the joint angles since they were calculated as relative degrees between attached body segments, within the same range of values for both sides. The six components of right medial and lateral KCFs comprised the output feature set, whereas the rest formed the input feature set. Features with negligible standard deviation (less than 1 × 10^−6^) were considered as noise and were omitted from the dataset. These included the lateral KCF across the x-direction, thereby ending up with 16 features for the input and 5 features for the output dataset (see Figure 1 and Table 2). 

The input data were stored in a 3D tensor X of size $N_{x}\;\times \;M\;\times \;T$, where $N_{x}=16$ is the number of input variables, M=4784 is the number of trials for all subjects, and T is the maximum number of time points. As the subjects walked with different speeds, some gait cycles are shorter than others and therefore the 3D tensor X is partially empty across its time dimension. We should underline that we do not temporally align the gait cycles because such an alignment can only been performed upon completion of the cycle. In an online prediction scenario where estimations should be provided for each time point, i.e., before the completion of the gait cycle, such an alignment is not feasible. The output data were stored in a similar way into a 3D tensor Y of size $N_{y}\;\times \;M\;\times \;T$, where $N_{y}=5$ is the number of output variables. Upon data splitting into training and testing sets (as described in Section 3.4), the sub-tensors (for both input and output variables) were unfolded and linearized so that each time measurement could be used as an independent sample for prediction. A description of all data variables used for prediction by machine learning is shown in Table 2. 

## 3. Prediction by Machine Learning

**Figure 2:**
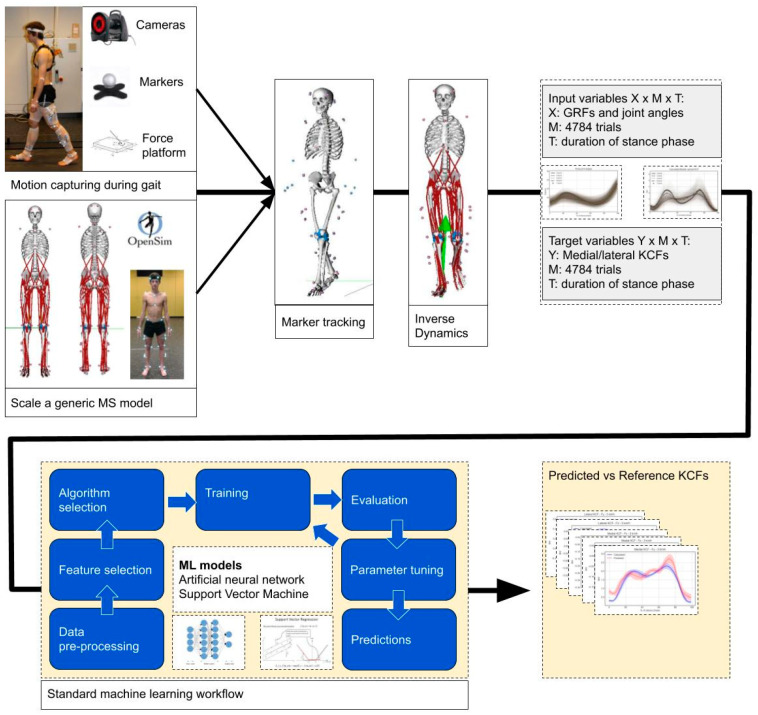
Our methodology for creation of the training set and development of the regression models.

In the next sections, we describe how the kinetic variables computed by the previous biophysics-driven modeling approach can be predicted using the fusion of kinematic variables and data-driven techniques. We formulate our problem as a regression problem and apply machine learning to solve it. For a complete overview of our methodology see Figure 2. 

### 3.1. Regression

Let us assume that f(x,w) is a family of functions parameterized by a vector w that transforms a vector x into a new space. As a note, we represent vectors as columns and use the superscript “*t*” to represent the transpose of a vector. Given a dataset of $N_{s}$ observations—namely, single time frames—$D=\left\{x^{i},y^{i}\right\},\;i=1,\ldots ,\;N_{s}$, where each observation i is expressed by an input vector ${\left(x\right)}^{t}=\left[x_{1},x_{2},\ldots ,x_{N_{x}}\right]$ with $N_{x}$ variables and a response variable (scalar) y, our goal is to estimate $\hat{w}$ that minimizes a loss function l between the transformed space representation $\hat{y}=f\left(x,\hat{w}\right)$ and the observed values for the $N_{s}$ training instances, i.e.,

(1) $$\hat{w}=argmin\sum_{i=1}^{N_{s}}l\left(f\left(x^{i},w\right),\;y^{i}\right)$$

As the true function that maps x into y (plus noise) is unknown, we will evaluate our results based on the residual of the predictions using the common root mean square error (RMSE) metric,

(2) $$RMSE=\;\sqrt{\frac{1}{N_{s}}\sum_{i=1}^{N_{s}}{\left(f\left(x^{i},\hat{w}\right)-y^{i}\right)}^{2}}$$

We examined two of the most popular methods for non-linear regression, the artificial neural networks [59] and the support vector regression [60]. Details on the transformation function and optimization mechanism for these two methods are provided next. 

### 3.2. Artificial Neural Networks 

ANNs have attracted the interest of the scientific community for classification of human movement [61,62] or prediction of meaningful biomechanical parameters [57] based on lower-level motion capture data, mainly owing to their significant ability to learn intermediate features over multiple levels of transformations. The generic structure a feed-forward ANN consists of multiple processor units, called neurons, organized in a series of layers including the first (input) layer, a set of intermediate (hidden) layers and the output layer. Each neuron is connected to the neurons of neighboring layers via numerical values (weights) and receives the weighted sum of all neuron outputs of the previous layer (except neurons from the input layer), after the application of an activation function. In our network, all the hidden neurons were activated using the “REctified Linear Unit–RELU” function according to the equation:

(3) f(x)=max(0,x),

The neurons of the output layer were fed with the weighted sum of all neurons’ outputs of the last hidden layer, which were activated by a linear function. Adaptive moment estimation (*Adam*) [63] algorithm was used to train the network by iteratively updating the weights, aiming at the minimization of the loss function, set as the mean squared error between the network predictions and the target values. To avoid overfitting, early stopping was utilized when a minimum loss was achieved and stabilized for 10 consecutive epochs. The total network topology was a four-layer ANN with two hidden layers of 400 neurons each, with the number of neurons in the input/output layers equal to the size of the input and target variables, respectively.

### 3.3. Support Vector Regression 

We investigated also support vector regression due to its high generalization ability, and because SVR optimization does not depend on the dimensionality of the input space, therefore is very well suited for datasets with a large number of independent variables. SVR, firstly proposed in [60], expands Vapnik’s concept of support vectors [64]. A basic (polynomial) transformation function for support vector machines can be expressed as

(4) $$f\left(x,w\right)=\sum_{i=1}^{N_{s}}\left(a_{i}^{*}-a_{i}\right){\left(x_{i}^{t}x+1\right)}^{p}+b,\;$$

where $x^{i}$ represents the *i*-th training instance, x a new input vector, and the $2N_{s}\;+\;1$ values of $a_{i}^{*},a_{i}$ and b form the vector w. The optimization problem of Equation (1) also entails regularization constraints over the magnitude of w to control the flatness of the solution. Moreover, a convex *ε*-insensitive loss function is adopted to only penalize predictions that are farther than *ε* from the desired output. 

Extending the original formulation of Equation (4) from linear, or polynomial, to non-linear mapping through the use of kernels, it is possible to achieve a higher accuracy if enough data are available to optimize for w and the kernel hyper-parameters. We chose a Gaussian (rbf) kernel with a fixed kernel scale of 0.6, avoiding optimization of hyperparameters. Since SVR does not support multi-output regression, five separate models were built for each target variable.

### 3.4. Assessment

The training and assessment of the regression models was performed in two different cross-validation settings. Firstly, we considered random data split over trials (*LeaveTrialsOut*), i.e., the trials of all subjects were divided in three equal parts and used to perform three-fold cross-validation, thereby allowing some trials from one subject to be in the training set and the other trials from the same subject in the test set. In the second setting, cross validation is performed across subjects (*LeaveSubjectsOut*), i.e., the subjects were divided in three equal parts such that all trials from each subject are either in the training set or in the test set. In both cases, the overall prediction accuracy was calculated by averaging the results over the three folds. Any method requiring tuning of hyperparameters exploited a small part of the training set only (to form a validation set), whereas the test set was left completely out for the final assessment of accuracy.

Assessment of the individual models trained for each KCF component was based on the error between predicted and calculated (based on musculoskeletal modeling) values. As the standard RMSE depends on the units, it impedes the results’ interpretation and comparison with others, if the data scale is unknown. Thus, we calculate the normalized RMSE (NRMSE), by dividing with the range of each variable (difference between maximum and minimum values) included in the dataset:

(5) $$NRMSE=\frac{RMSE}{\Delta y}*100\%$$

where RMSE is the root mean squared error calculated in the original range of values, and Δy is the range of values for each KCF component. Τhe Δy values used for the results of this paper were 8.069, 12.164, 1.389, for the anteroposterior, distal proximal and mediolateral medial KCF, and 9.657, 0.007 for distal proximal and mediolateral lateral KCF, respectively.

Although the RMSE is the most representative metric for prediction accuracy, it is sensitive to outliers and to systematic errors such as baseline shifts. Therefore, we also calculated the Pearson’s correlation coefficient (R) as a measure of association strength between predicted and calculated values, which is less sensitive to outliers. 

## 4. Results

### 4.1. Calculated KCFs Based on Musculoskeletal Modeling

**Figure 3:**
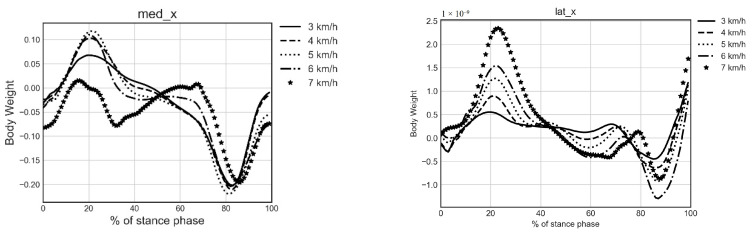

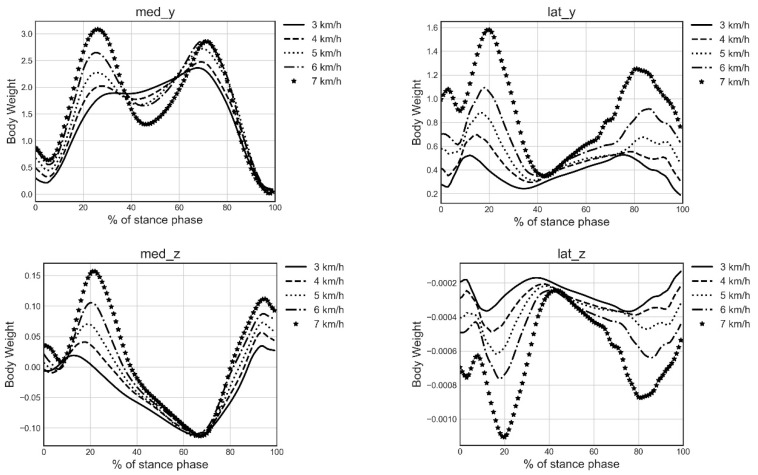
Ensemble curves of the 6 components of medial and lateral KCFs along the stance phase.

All (M = 4784) trials were successfully processed in OpenSim using standard inverse dynamics and a dedicated musculoskeletal model as described in Section 2.3. To demonstrate the validity of the calculated KCFs and deem their suitability for training ML models, respective average curves and their peak values were calculated and compared with literature. Individual gait cycles were identified, and all data were time normalized to 100 points by quadratic interpolation. An averaging process of knee forces per gait cycle within and between subjects for each speed resulted in the ensemble curves for each component of medial and lateral KCFs, as shown in Figure 3. The resulting ensemble curves were calculated separately along the three directions, i.e., in the anteroposterial direction (*x*), distal–proximal direction (*y*) and mediolateral direction (*z*). Positive *x* values indicate backwards, positive *y* values downwards and positive *z* inwards. 

Moreover, a double peak profile was found in both medial and lateral vertical components (med_y and lat_y), with first and second peak occurring at early and late stance phase, respectively. Mean peak KCF values were calculated for each force component by averaging identified first and second peak values for every gait cycle per speed, within and between subjects. Results (see Table 3) show that first and second peak of med_y increase with increasing speed, ranging from 2.18 to 3.30 body weight (BW) and 2.50 to 3.22 BW, respectively. The same finding applies to the first and second peak of lat_y, ranging from 0.71 to 1.84 BW and 0.70 to 1.73 BW, respectively. 

### 4.2. ML-Based Prediction with and without GRFs

**Figure 4:**
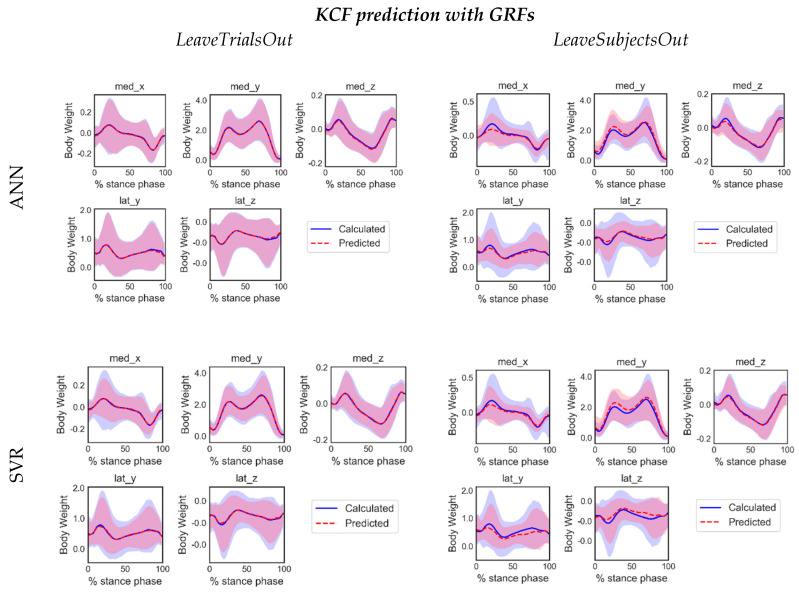
Ensemble curves along the duration of the stance phase for the 5 different components of knee joint forces computed by inverse dynamics (in blue) and predicted by ANN and SVR based on LeaveTrialsOut and LeaveSubjectsOut training scenarios. The graphs illustrate the distribution of time normalized testing trials of one (out of three) fold summarized by the mean curve (solid line) and range of variation (5–95% percentile of the distribution).

We chose to train ML models with two versions of inputs, i.e., with and without GRFs, on grounds of limited availability of GRF-measuring equipment in usual mocap settings. The ANN and SVR predictions were compared against calculated KCFs. Generally, ANN slightly outperformed SVR in most cases, both in terms of NRMSE and R. Average-across-folds values of NRMSE and Pearson’s R for every model with or without GRFs, are depicted in Table 4 and Table 5, respectively. Mean NRMSE values for ANN-GRF were smaller and *R* values larger than the corresponding from SVR-GRF. The same trend occurs when only joint angles are included in the input set: mean NRMSE values for ANN-noGRF were smaller than the corresponding values for SVR-noGRF, while R was mostly larger for both *LeaveTrialsOut* and *LeaveSubjectsOut* cross-validation settings. No substantial differences were noticed between the prediction accuracies of GRF and noGRF models per case, thus ensemble curves across trials of predictions and calculated KCFs were illustrated only for models using GRF, as depicted in Figure 4. 

A review of previous outcomes on predictive capability of different ML models for KCFs is summarized in Table 6. 

### 4.3. Robustness of Fit

**Figure 5:**
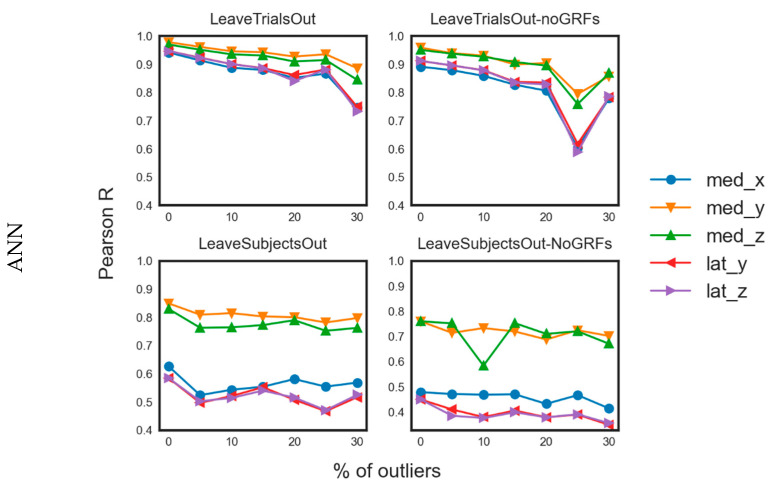
Line charts for the *R* values related to the predictions of the different models on test data with different levels of data contamination by outliers.

Results from musculoskeletal modeling methods are highly sensitive to instantaneous error registration during motion capture, due to a faulty marker recording or noisy IMU signal. Although low pass filtering techniques can remove minor drifting and ensure smoothness of signals [67], joint force calculation remains susceptible to highly erroneous recording settings, propagating inconsistencies in equations of motion, thus abnormal values can infiltrate the training set. To evaluate the robustness to outliers of the proposed ML models, we selected the best performing model (i.e., the ANN) and examined its sensitivity to approximate the target variable with varying amount of introduced noise. Specifically, we artificially contaminated our dataset with outliers, generated by sampling from a Student-t distribution with zero mean and 2 degrees of freedom and scaled by the standard deviation of the original training data. The original samples were randomly replaced at proportions of 5%, 10%, 15%, 20%, 25% and 30% of the training samples [68]. Training of the ANN with the contaminated datasets was similar as before, and average values between folds were calculated for each target variable. Average values of *R* across folds for every contamination level are shown in Figure 5. As expected, increasing presence of outliers in the training set reduced the predictive capability of the ANN under different training scenarios, although not significantly in most cases, indicating its high prediction robustness, as also observed in previous work [69]. 

## 5. Discussion

The current study demonstrates for the first time a mocap-agnostic, ML-empowered framework for prediction of multi-component KCFs during different speeds of walking and showcases the advantages of using supervised learning coupled with musculoskeletal modeling in mapping the kinematic to joint force space. Multi-output regression enabled us to leverage correlations between the different KCF components and improve the predictive accuracy rather than depending on separate regressions that could overlook intra-component relation [70]. The prediction capability of artificial neural network and support vector regression models was examined under different training schemes with two levels of exposure to the dataset, identifying ANN as the best ML method among the investigated ones. Last, testing different model configurations with artificially contaminated training sets with outliers in different proportions indicated that ANN can successfully approximate the nonlinear function mapping joint angles with or without GRFs to KCFs, even when errors are present in the measurements.

Reference values of inter-compartmental peak KCFs in different speeds during walking are of significant importance for scientists exploring the dynamic loading of the musculoskeletal system and its effects on local tissue response. Resultant and vertical forces are rarely distinguished in the literature, probably because of the high similarity between their magnitudes since mediolateral and anteroposterior components show negligible values. Nonetheless, the current work presents the three components separately for a complete overview of healthy knee loading milieu. Average medial and lateral force values for both peaks during stance phase fit well within the range of other modeling studies or in vivo data, despite the large differences in cohorts’ profile or modeling practices of previous work, as reviewed from Fregly et al. [71]. Few studies report peak values in certain gait speeds. Specifically, Zhao et al. [72] reported in vivo medial peak forces of 1.51, 1.58 BW and 1.63 BW at speeds of ~3, 4.5 and 5.5 km/h of one total knee arthroplasty (TKA) patient during gait, that are similar to the lower bounds of our estimations. Our results also compare favorably with mean peak medial knee forces of 2.08 BW and 2.25 BW as reported by Kutzner et al. [73], for the first and second peak, respectively, at an average gait speed of ~4 km/h from three TKA patients. Regarding modeling efforts, most studies focused on building sophisticated subject-specific knee models to reproduce in vivo loads from TKA patients, leaving loading of the healthy knee mostly unexplored. Winby et al. [74] first introduced a computationally efficient way to estimate inter-compartmental forces of healthy gait, reporting—similar to ours—peak medial and lateral values of 2.0–3.0 BW and 1.0–1.4, respectively, during level walking. Kumar et al. [75] also reported comparable average values from a healthy elderly cohort, namely, 2.37 and 1.80 BW for the first and second peak of medial KCFs, respectively, along with 1.30 and 0.45 BW for the first and second peak of lateral KCFs, at the walking speed of 5.6 km/h. In conclusion, the current study has provided valid results of healthy knee loading on treadmill walking, providing previously unexplored medial and lateral forces on a wide range of speeds. 

Our prediction results showed that multidimensional medial and lateral KCF can be sufficiently predicted by ANN or SVR based on joint angle data—with or without GRFs—although ANN performed better in both cases. The prediction errors were determined in respect to the level of exposure to the full dataset, being low when the model was partially exposed to some trials of the testing subject (*LeaveTrialsOut*) and relatively high when no information from the testing subject was used during training (*LeaveSubjectsOut*). The implemented ANN achieved a good to excellent prediction score in the case of the compressive medial KCF in the distal–proximal (vertical) direction regardless of training scenario, which is of significant importance since that force component is the main determinant of medial knee loading and is considered an important risk factor for local cartilage degeneration. This finding can be attributed to its steady-across-trials double peak profile and the resulting similarity between training and test distributions. Hence, the regression model managed to sufficiently map inputs and outputs and achieve good estimation accuracy, even when the training was artificially contaminated with outliers in different amounts. Although comparisons between studies are problematic due to variations in datasets and methods, the present study generally outperformed earlier efforts to predict medial KCFs (see Table 6), possibly the main reason being the extensive dataset used in this study and the range of speeds or shod conditions incorporated, thus substantial pattern variation was induced. Based on comparisons of *R* values between our method and Ardestani et al.’s [48] (see Table 6), inclusion of subject-specific EMGs in the training set by the latter seem to enhance model predictive power in the *LeaveSubjectsOut* scenario, yet, respectively, a higher NRMSE% may indicate limitations in that approach. Moreover, EMG acquisition requires advanced equipment and high expertise, which is impractical in common clinical settings, in contrast with our approach that provides prediction of KCFs solely based on joint angles estimation, irrespective of the mocap system used. Hence, IMUs or even video-based mocap systems that are inexpensive, portable and can offer real-time joint angle estimations, are appropriate for providing the necessary input to our trained models. 

Different models’ exposure levels to the dataset revealed that their generalization capability was compromised when all the trials of the testing subjects were excluded from the training dataset, which is in accordance with previous findings [47,48]. Although ANN’s predictive power of medial KCF was only slightly diminished in the *LeaveSubjectsOut* setting, it reaches moderate prediction level for lateral KCFs and medial anteroposterior force. This can be partially explained by the lack of consistent relationship between kinematic and kinetic variables in the lateral KCF or the noise level is a serious confounding factor that hinders the discovery of any underlying relationship. Additionally, uniqueness of individual joint motion characteristics during gait [69] may be an additional challenge when the models are tested in data of unseen subjects. Strategies to overcome such limitations may include larger datasets from larger cohorts or appropriate data augmentation techniques to induce adequate variability within the training set, along with deeper architectures allowing a higher degree of non-linearities. 

The current study outlines the possibility to use only kinematic variables to predict KCFs during walking, as indicated from the high prediction accuracy of the trained models when GRFs were excluded from the training set. Such redundancy may be indicative of the dissociation between GRFs and joint loading found in earlier studies [76,77], pinpointing kinematics and associated overall motion strategy as most relevant. Using only kinematics for KCF estimation seems promising in all cases when synchronous joint angle estimation and GRF acquisition is not feasible due to equipment/space restrictions. For instance, IMU-based on-field measurements could be utilized in combination with our model to explore outdoors’ mobility in terms of knee loading, and thereby gain more insight to real-world gait dynamics in environments inappropriate for dedicated mocap equipment. Within the same context and in contrast with standard physics-based modeling, the ML-empowered method requires no further knowledge on muscle physiology and overall anatomy, outlining its attractiveness for KCF prediction in real time. 

Limitations of the current study mainly lie on the development of a rich and representative training dataset, a task extremely challenging in the context of musculoskeletal modeling. Owing to lack of in vivo data, especially in healthy populations, knee forces had to be calculated under a rigorous and time-consuming modeling framework, yet inherently limited to the subject sample, motion capture protocol, and simulation process. Calculation of KCFs require sophisticated musculoskeletal models, nonetheless, being a mere approximation of the actual human function, hence affecting their calculation. No subject-specific information on knee geometry was available, hence overestimation of actual knee forces might have infiltrated our results, as shown in [58], where estimated medial knee forces were compared between a generic (uninformed) knee model and a (fully informed) model with known contact point distance and tibiofemoral alignment, extrapolated from medical images. Our future plans include the exploitation of previous work of our group on the automatic generation of subject-specific and anatomically adopted knee geometry meshes [78] that allow one to personalize the KCF computations, if imaging information is available. Additionally, the reported forces in the anteroposterial and mediolateral directions must be cautiously considered, since no mathematical formulation is incorporated in the knee model that allows the sharing of the prescribed loads between the two knee contact points in the respective planes of motion. Hence, infinite couples of individual values could sum up correctly to the net reaction forces. A systematic analysis of the synergetic nature of the joint is a challenging direction for future work, as an extension of similar work for muscle synergies found in [79]. Moreover, ANN prediction accuracy relies heavily on the network topology. As a costly brute-force search allowed us to examine only a few parameter values, detailed fine-tuning in search of the optimal settings may have resulted in better results. Nonetheless, high correlation coefficients and low NRMSE support the validity of the selected parameters. Last, the choice of joint angles as inputs (versus marker trajectories) was made upon the rationale that KCF prediction should be independent of motion tracking system, so that the trained ML models could be used in various settings. However, this introduces the need to calculate the joint angles (via inverse kinematics) prior to KCF estimation. Although this is an easy and computationally fast processing step, the introduced biomechanical modelling procedure might insert more variability than the variability introduced by the differences in markers’ placement. Therefore, we cannot be sure if the original input data (marker coordinates) can lead to improved prediction power of the used ML models. A follow-up study may address this issue.

Our study showed that integration of advanced musculoskeletal modeling techniques and artificial intelligence algorithms proved to be successful in predicting knee joint loading profiles during gait stance phase for different walking speeds. The selected ML model was able to predict multi-component KCFs instantaneously relying solely on motion-specific joint angles, also outlining the redundancy of GRF data. In addition, prediction of medial and lateral KCF components was in good agreement with calculated data, especially in the case of clinically relevant medial vertical KCF, highlighting the potential of machine learning approaches in contrast with standard musculoskeletal modeling techniques. 

Predictive power was compromised in the case of lateral KCFs and especially for the *LeaveSubjectOut* scenario, indicating that inclusion of subject-specific motion data in the training set seems imperative to achieve the highest prediction accuracy within the current methodology. This imposes a hindrance in the analysis since it requires the acquisition of new data and model re-calibration every time a new subject is going to be assessed. Thus, future work should address the need for more sophisticated machine learning models and architectures to approximate more abstract kinematic to kinetic variables’ relationship and attain acceptable prediction rates of important variables for previously unseen subjects. 

Finally, our method can be extended to real-time prediction of other motions and loading variables solely based on joint angles, such as hip/ankle joint, ligament or muscle forces, once provided with the appropriate dataset and musculoskeletal modeling workflow. Thus, well-informed decision making will be facilitated for clinical settings with limited equipment, enabling medical professionals to explore real-world motion dynamics for the design of targeted training protocols, or subject-specific rehabilitation programs. For example, the current methodology can be implemented in a framework that can train a professional athlete to perform landings in a joint-protective fashion to avoid injury while exercising in an athletic environment or seamlessly guide osteoarthritic patients to offload joint structures and address local cartilage degeneration during outdoor activities. 

## 6. Conclusions

This paper presents the use of machine learning techniques to obtain surrogate models of biophysics-driven simulations for real-time biomechanical computations. Such ML-empowered models can be used whenever the original computational modelling approaches are too slow or resource demanding. Their application can be combined with finite element analyses or augmented reality technologies to explore force-driven, local bone deformation levels or facilitate balance and mobility rehabilitation and support treatment strategies for gait alteration for pathological populations. Integration of such trained models in digital tools will augment clinical practice and evidence-based decision making. Seamless streaming of important scientific information via advanced visualization interfaces or game-like platforms in real world settings, will enhance user experience and engage involved parties, however, meticulous collection and processing of input data from portable devices for offline or real-time analysis remains a challenge. Our results indicate the high potential of the ANN model even for recordings of new subjects, while the SVR model requires the incorporation of previous trials of the test subject for stable performance. In the future, we plan to investigate also other machine learning techniques, such as convolutional neural networks with sparse coding for acceleration [80], and graph neural networks [81]. 

## Figures and Tables

**Table 1:** Participant characteristics.

No. Subjects		Mean Age (SD) (Years)		Body Mass Index (kg/m^2^)		Percent Female	
Young 40	Elderly 14	Young 22 (1.66)	Elderly 69.6 (3.5)	Young 22.8 (2.8)	Elderly 24.4 (2.3)	Young 60%	Elderly 100%
Total = 54							

**Table 2:** Input and target tensors characteristics.

Anatomical Location	Abbreviation	Component	Units
torso (lumbrosacral joint)	lumbar	extension bending rotation	degrees
pelvis	pelvis	tilt list rotation	
hip joint	hip	flexion adduction rotation	
knee joint	knee	flexion	
patella knee angle	patella	flexion	
ankle joint	ankle	flexion	
subtalar joint	subtalar	eversion	
**Force Description**			
ground reaction force	GRF	anteroposterior (*x*) distal proximal (*y*) mediolateral (*z*)	body weight (BW)
medial knee contact force	KCF (med)	anteroposterior (*x*) distal proximal (*y*) mediolateral (*z*)	
lateral knee contact force	KCF (lat)	anteroposterior (*x*) distal proximal (*y*) mediolateral (*z*)	

**Table 3:** Mean peak vertical medial and lateral knee contact forces (standard deviation) during different gait speeds.

	Medial Force		Lateral Force	
	1st Peak	2nd Peak	1st Peak	2nd Peak
3 km/h	2.18 (0.58)	2.50 (0.75)	0.71(0.34)	0.70 (0.39)
4 km/h	2.22 (0.58)	2.76 (0.82)	0.92 (0.45)	0.81 (0.49)
5 km/h	2.48 (0.63)	3.06 (0.86)	1.15 (0.49)	1.02 (0.49)
6 km/h	2.82 (0.71)	3.25 (0.96)	1.44 (0.63)	1.36 (0.79)
7 km/h	3.30 (0.65)	3.22 (1.05)	1.84 (0.68)	1.73 (0.77)

**Table 4:** NRMSE (%) for medial (med) and lateral (lat) KCF components in *x*, *y* and *z*.

	med_x		med_y		med_z		lat_y		lat_z	
*LeaveTrialsOut*										
	***GRF***	*noGRF*	***GRF***	*noGRF*	***GRF***	*noGRF*	***GRF***	*noGRF*	***GRF***	*noGRF*
SVR	1.04	1.36	2.68	3.92	2.03	2.65	2.80	3.44	2.79	3.44
ANN	0.67	0.9	1.71	2.35	1.45	1.82	1.61	2.04	1.61	2.04
*LeaveSubjectsOut*										
	***GRF***	*noGRF*	***GRF***	*noGRF*	***GRF***	*noGRF*	***GRF***	*noGRF*	***GRF***	*noGRF*
SVR	1.53	1.73	4.63	5.85	3.42	3.80	4.41	4.66	4.41	4.65
ANN	1.60	1.81	4.54	5.39	3.49	3.85	4.19	4.59	4.19	4.59

**Table 5:** *R* for medial (med) and lateral (lat) KCF components in *x*, *y* and *z* direction.

	med_x		med_y		med_z		lat_y		lat_z	
*LeaveTrialsOut*										
	***GRF***	*noGRF*	***GRF***	*noGRF*	***GRF***	*noGRF*	***GRF***	*noGRF*	***GRF***	*noGRF*
SVR	0.85	0.73	0.94	0.88	0.94	0.89	0.83	0.73	0.83	0.73
ANN	0.94	0.89	0.98	0.96	0.97	0.95	0.95	0.91	0.95	0.91
*LeaveSubjectsOut*										
	***GRF***	*noGRF*	***GRF***	*noGRF*	***GRF***	*noGRF*	***GRF***	*noGRF*	***GRF***	*noGRF*
SVR	0.64	0.50	0.83	0.73	0.82	0.77	0.52	0.44	0.52	0.44
ANN	0.63	0.48	0.85	0.76	0.83	0.76	0.58	0.45	0.58	0.45

**Table 6:** Review of machine learning (ML)-based KCF prediction methodologies.

	Subjects	Test Trials	Classifier	Inputs	Y ^2^	Mean Pearson’s R (NRMSE%) ^3^	
						LeaveTrialsOut	LeaveSubjectsOut
Ardestani et al. (2014) [48]	4 (knee replacement patients)	75	ANN	-GRFs -marker 3D coordinates -EMG	in vivo	0.96 (10.5)	**0.94** (13.3)
Rane et al. (2019) [47]	healthy and knee OA patients	58 63 28	CNN	-CoP ^1^ -GRFs -marker 3D coordinates	ID	0.90	0.90 0.87
Stetter et al. (2019) [65]	13 healthy athletes (young)	198	ANN	-2 IMUs	ID	-	0.87
Zhu et al. (2019) [66]	3 (knee replacement patients)	135	Random Forest	-GRFs -marker 3D coordinates -EMG	in vivo	0.97	-
**Proposed method**	**54 healthy (young and elderly)**	**4784**	**ANN**	**-GRFs** **-Joint angles**	**ID**	**0.98** **(1.71)**	0.85 **(4.54)**

^1^ CoP: center of pressure. ^2^ Y: ML model trained on inverse dynamic (ID) modelling or instrumented knee prosthesis (in vivo) data. ^3^ For fair comparison, the accuracy is reported only for the common (across studies) target variable, i.e., vertical KCF.
